# Routine use of 16S rRNA PCR and subsequent sequencing from blood samples in septic shock: about two case reports of *Capnocytophaga canimorsus* infection in immunocompetent patients

**DOI:** 10.1186/s12879-022-07328-z

**Published:** 2022-04-09

**Authors:** Alexandre Martins-Baltar, Sylvain Meyer, Olivier Barraud, Fabien Garnier, Marie-Cécile Ploy, Philippe Vignon, Bruno François

**Affiliations:** 1 Réanimation Polyvalente, CHU Dupuytren, 2 Avenue Martin-Luther King, 87042 Limoges, France; 2 Laboratoire de Bactériologie-Virologie-Hygiène, CHU de Limoges, Limoges, France; 3 UMR Inserm 1092, Faculté de Médecine, Limoges, France

**Keywords:** *Capnocytophaga canimorsus*, Septic shock, 16S rRNA PCR, Dog bite, Diagnosis, Sequencing

## Abstract

**Background:**

*Capnocytophaga canimorsus* infection happens frequently in immunosuppressed patients with reported domestic animal bites. Clinical presentation ranges from simple cellulitis to fulminant septic shock with disseminated intravascular coagulopathy, with an overall mortality of 30%. Conventional blood culture is often negative as this is a slow-growing pathogen. Nevertheless, the increasing use of 16S rRNA gene amplification and Sanger sequencing allows a much more rapid diagnostic confirmation. We present two case reports where 16S rRNA gene sequencing helped to diagnose *Capnocytophaga canimorsus* infection.

**Case presentation:**

Case 1: A 53-year-old man with a history of non-cirrhotic chronic alcohol consumption was admitted to the intensive care unit (ICU) for septic shock and disseminated intravascular coagulopathy (DIC) of unknown origin. Blood cultures remained negative and a 16S rRNA PCR was performed leading to the identification of *Capnocytophaga Canimorsus* on day 4*.* Targeted antibiotic therapy with ceftriaxone for 14 days lead to overall recovery. Afterwards, the patient recalled a dog bite 2 days before hospitalization with a punctiform necrotic wound localized on a finger, which was not obvious at admission. Case 2: A 38-year-old man arrived to the emergency department for acute alcohol intoxication and history of a dog bite 2 days before. At admission, septic shock with purpura fulminans was diagnosed and required ICU hospitalization, invasive mechanical ventilation, vasopressor support and renal replacement therapy due to the rapid clinical deterioration. In the context of septic shock with purpura fulminans, DIC and recent dog bite, the diagnosis of *Capnocytophaga canimorsus* septic shock was suspected, and early confirmed by 16S rRNA PCR coupled to Sanger sequencing on day 2. Blood cultures became only positive for *Capnocytophaga canimorsus* 5 days after admission*.* Ceftriaxone alone was infused for 10 days in total, and the patient was discharged from the ICU on day 25.

**Conclusions:**

16S rRNA gene PCR proves an important diagnostic tool when facing a sepsis of unknown origin. In these two cases of septic shock related to *Capnocytophaga canimorsus*, initial blood cultures remained negative at 24 h, whereas the diagnosis was achieved by 16S rRNA PCR sequencing performed from blood samples obtained at admission.

## Background

*Capnocytophaga canimorsus* is an uncommon pathogen, which might lead to life threatening infections in humans. It is an oral commensal fusiform Gram-negative bacillus mainly found in cats and dogs [1]. Cases occur in patients with reported domestic animals, with or without bite history [2], and frequently in a context of secondary immunosuppression such as asplenia, chronic steroid treatment, alcoholism or cirrhosis [3]. Symptoms can appear one to seven days after inoculation [4] with a wide clinical presentation ranging from a simple cellulitis to fulminant septic shock with disseminated intravascular coagulopathy (DIC). Mortality is estimated between 25 and 30% [5]. This high rate can be explained by the strong virulence of *C. canimorsus* caused by its abilities to escape phagocytosis [6] as well as its gliding motility allowing easier diffusion towards general circulation. *C. canimorsus* belongs to the family of slow-growing bacteria and may not be identified using conventional blood cultures sometimes [7], thus causing deleterious diagnostic wandering.

Regarding the low prevalence of such infections, no targeted tool exists. The 16S rRNA gene amplification and Sanger sequencing is a useful approach to detect bacteria directly from normally sterile body fluids. Even if it is not widely implemented, it is more and more routinely used in clinical microbiology laboratories to identify the bacteria that are difficult to isolate using standard culture methods [8].

In the present report, we discuss two cases of septic shock related to *C. canimorsus*, in which initial blood cultures remained negative at 24 h, whereas the diagnosis was achieved by 16S rRNA PCR sequencing performed from blood samples obtained at admission.

## Case presentation

### Case # 1

A 53-year-old man presented to the emergency department (ED) with fever, diarrhea and abdominal pain. His medical history included meningitis at 3 years old, amebiasis and alcohol consumption without cirrhosis. Physical examination at admission was remarkable for fever (40 °C core temperature), low blood pressure (88/55 mmHg), tachycardia (140 bpm), upper right abdominal pain, marbling and fulminant purpura with petechial bleeding and necrotic toes. Abdominal CT scan performed at admission was normal. The first laboratory results evidenced compensated metabolic acidosis (pH: 7.44; PaCO_2_: 24 mmHg; base excess: − 7 mmol/L) with hyperlactatemia (6.1 mmol/L), acute renal failure (serum creatinine: 149 µmol/L) and rhabdomyolysis (CK: 7600 UI/L), leukopenia (3.96 G/L) with lymphopenia (0.15 G/L), and thrombocytopenia (27 G/L) together with a low prothrombin ratio (48%), confirming a DIC. Blood cultures were drawn (aerobic and anaerobic bottles) and incubated using the BacT/ALERT3D System (bioMérieux, Craponne, France).

Septic shock was diagnosed and the patient was transferred to the intensive care unit (ICU). Transthoracic echocardiography showed a septic cardiomyopathy with biventricular dysfunction (left ventricular ejection fraction = 20%). The hemodynamic condition of the patient deteriorated despite resuscitation with 2 L of saline and vasopressors were started with norepinephrine (NE) up to 0.8 µg/kg/min and dobutamine at 5 µg/kg/min. In the absence of obvious source of infection, empirical antibiotic treatment was initiated early with ceftriaxone, clindamycin and metronidazole. In the absence of positivity of the blood cultures after 48 h, a 16S rRNA PCR was performed on a blood sample obtained at admission and used for complete blood count. Total DNA was extracted from the EDTA vial using the SaMag-12® automaton (Sacace Biotechnologies). A SYBR-green qualitative PCR on SmartCycler® (Cepheid) targeting a part of the 16S rRNA gene was applied to the DNA extract using the 91E and the 13BS primers yielding a 492-bp fragment [9]. This sequence targets the V3-V4 region of the 16SrRNA that enables adequate differentiation for identification and can provide a bigger percent difference between strains than the entire 16S sequence [10]. 16S rRNA PCR amplicons were purified and sequenced on the 3500 XL (Applied Biosystem) Sanger sequencer. Sequences were compared with the GenBank database using the BLASTN analysis leading to the identification of *C. canimorsus* with a 100% identity score*.* Bacterial identification was obtained 4 days after admission. The antibiotic treatment was adjusted to ceftriaxone alone for a total of 14 days. Blood cultures remained negative after the 5-day incubation. The patient slowly improved and was weaned off NE at day 2. The main sequela was a distal dry gangrene of multiple toes and the patient was transferred to a rehabilitation unit after 25 days of hospital stay. Afterwards, the patient recalled a dog bite 2 days before hospitalization with a punctiform necrotic wound localized on a finger and which was not obvious at admission.

### Case # 2

A 38-year-old man presented to the ED with confusion, emesis and abdominal pain in a context of acute alcohol intoxication for 3 days. He had no past medical history. At admission he had an unstable hemodynamic status with tachycardia (130 bpm), low blood pressure (90/53 mmHg), lower limbs and abdominal marbling, and extensive purpura fulminans. He also presented mild hypothermia (36.4 °C), polypnea (respiratory rate at 40/min) and desaturation (86% under 15 L/min of O_2_ with bag mask). The patient reported a dog bite 2 days before admission. Laboratory results evidenced a partially compensated metabolic acidosis (pH: 7.22; PaCO_2_: 17 mmHg; base excess: − 18 mmol/L), very severe hyperlactatemia (18 mmol/L), hyperleukocytosis (20 G/L) without lymphopenia, DIC with thrombocytopenia (7 G/L) and low prothrombin time (53%), and acute renal failure (serum creatinine: 354 µmol/L). Septic shock with purpura fulminans was diagnosed and the patient received 2 L of fluid resuscitation and empirical antibiotic therapy with ceftriaxone, gentamicin and metronidazole before being transferred to the ICU. Considering the rapid deterioration of his general status, the patient was intubated and mechanically ventilated. His hemodynamic condition deteriorated and required up to 0.24 µg/kg/min of NE. In the context of septic shock with purpura fulminans, DIC and recent dog bite, the diagnosis of *C. canimorsus* septic shock was suspected. In this context, a 16S rRNA PCR coupled to a Sanger sequencing (same protocol as in case#1) was performed on the blood sample obtained at admission and confirmed *C. canimorsus* within 36 h of admission. Accordingly, Ceftriaxone alone was infused for a total of 10 days. Blood cultures became positive 5 days after admission and yielded a *C. canimorsus* strain. An antibiotic susceptibility test was performed. The result was only obtained after the initiation of the treatment and the ongoing antibiotic therapy was continued. NE was weaned after 2 days. Because of a kidney failure, the patient needed renal replacement therapy and was transferred to the nephrology department after 25 days of ICU stay and discharged home after 54 days of hospitalization. The main sequela was a distal dry gangrene of multiple toes.

## Discussion and conclusions

A critical aspect of sepsis management is the rapid identification of the pathogen to confirm the appropriateness of antibiotic therapy. This is even truer when considering some very aggressive bacteria such as *C. canimorsus*, because of its high morbidity and mortality. When facing clinical scenarios in the absence of etiologic diagnosis, clinicians usually prescribe empirical combined therapy with broad-spectrum antibiotics. Blood cultures failed to identify the pathogen in the first case and only became positive after 5 days in the second case, exposing patients to unnecessary broad-spectrum antibiotic therapies, with the risk of associated side effects including the selection of multidrug resistant bacteria or additional renal toxicity.

*Capnocytophaga canimorsus* is a slow-growing bacterium that can be difficult to cultivate. Thus, the use of 16S rRNA gene PCR and sequencing directly from blood samples remains one of the best strategies to identify bacteria in the context of septic shock with negative blood cultures. Although the 16S rRNA technology has a comparatively higher sensitivity for identification of non-cultivable bacteria, its use is limited to single bacterial infections. Therefore, this technique remains of high interest in rare and hard-to-diagnose diseases. 16S rRNA PCR has also shown good diagnostic results on other types of samples such as cerebro-spinal fluids or cardiac valves [7, 11]. Fast next generation sequencing strategies are being developed and will probably enable more rapid and accurate identification of bacteria [12], even though their implementation in clinical laboratories for routine diagnosis is still difficult [13].

16S rRNA PCR and sequencing is a supporting tool to diagnose infections, which is routinely used in an increasing number of laboratories. This technique is first able to confirm a bacterial origin of the septic case and has to be better considered in case of septic shock with negative blood cultures in order to early document slow-growing bacteria such as *C. canimorsus* and adapt the initial empirical antibiotic therapy.

## Data Availability

The datasets used and presented in this paper have not been published but are available from the corresponding author on reasonable request.

## Abbreviations

DIC: Disseminated intravascular coagulopathy
DNA: Deoxyribonucleic acid
ED: Emergency department
EDTA: Ethylene diamine tetra-acetic acid
ICU: Intensive care unit
NE: Norepinephrine
PCR: Polymerase chain reaction
RNA: Ribonucleic acid
